# Evolution of a functional taxonomy of career pathways for biomedical trainees

**DOI:** 10.1017/cts.2018.22

**Published:** 2018-08-08

**Authors:** Ambika Mathur, Patrick Brandt, Roger Chalkley, Laura Daniel, Patricia Labosky, Constance Stayart, Frederick J. Meyers

**Affiliations:** 1 Graduate School, Office of Workforce Training, Development and Diversity, Department of Pediatrics, Wayne State University, Detroit, MI, USA; 2 Career Development and Training, Office of Biomedical Graduate Education, University of North Carolina, Chapel Hill, NC, USA; 3 Office of Biomedical Research Education and Training, Nashville, TN, USA; 4 Broadening Experiences in Scientific Training, Office of Biomedical Research Education and Training, Vanderbilt University School of Medicine, Nashville, TN, USA; 5 Office of Strategic Coordination, Division of Program Coordination, Planning, and Strategic Initiatives, Office of the Director, National Institutes of Health, Bethesda, MD, USA; 6 myChicago Options In Career Empowerment, University of Chicago, Chicago, IL, USA; 7 Department of Internal Medicine, Precision Medicine, University of California Davis School of Medicine, Sacramento, CA, USA

**Keywords:** Biomedical career outcomes, career pathways, taxonomy, research career development, STEM

## Abstract

Several reports have shown that doctoral and postdoctoral trainees in biomedical research pursue diverse careers that advance science meaningful to society. Several groups have proposed 3-tier career taxonomy to showcase these outcomes. This 3-tier taxonomy will be a valuable resource for institutions committed to greater transparency in reporting outcomes, to not only be transparent in reporting their own institutional data but also to lend greater power to a central repository.

## Introduction

The national conversation about the training experiences of doctoral and postdoctoral scholars and a timely programmatic response to workforce needs has been hindered by the lack of a reliable data set based upon a taxonomy of the diverse career outcomes scholars might pursue. The recommendation to reduce the number of trainees is based on the declining numbers of tenure-track faculty positions in research institutions [1] and may not consider that there are other workforce needs. Labor market shifts may not account for the loss of interest in academic careers [2]. The training and mentoring community as a whole has not aggregated, analyzed, and disseminated information about career outcomes that could guide trainee career and professional development programs and could inform and impact faculty opinions.

The absence of a unified taxonomy is reflected in many aspects of the biomedical research ecosystem. First, historically, many academic institutions and federal funding agencies have prioritized the importance of Ph.D. scientists who intend to enter an academic career. A survey from 39 institutions of 4100 Ph.D. students in life sciences, chemistry, and physics reported that students felt strongly encouraged by their advisors to pursue research careers (primarily in academia) [3]. Many academic institutions have not prepared their trainees for careers other than the traditional role as a principal investigator in academia [4–6]. Similarly, federal training grants, such as the National Institutes of Health (NIH) T32 mechanism, reinforced this mindset through their review criteria of applications. Trainees considering careers other than academia were reluctant to seek mentoring and career development for fear of being considered failures. The NIH T32 mechanism review criteria have been amended in the most recent call to include nonacademic research careers as important outcomes of training grants.

Second, trainees have pursued diverse careers despite the absence of institutional support. Tilghman and her colleagues reported that less than 25% of trainees pursue principal investigator careers [7] confirmed by Fuhrmann *et al*. [8] and others [9].

Third, trainee dissatisfaction has been significant due to a lack of training in transferable skills, such as organizational and leadership management, team project management, budget planning, and career development skills [10]. Trainees without guidance are left with a sense of abandonment toward their degree-granting institution.

Fourth, many institutions that have tried to introduce funding for professional and personal development have done so in an environment unsupportive of such endeavors. Academic institutions face the increasing need for talented trainees to advance biomedical research and operate within the long-extant infrastructure of biomedical research training that has never recognized the need for trajectories that lead outside of the academic environment. These issues have prompted a broader conversation in the biomedical graduate training community [7, 11]. Trainees have been increasingly advised to take an active role in exploring their own career options either early in, or even before, they start a Ph.D. program [9, 12], frequently without institutional structures and resources.

Responding to this national need, in 2013 the NIH Common Fund issued a Funding Opportunity Announcement for the “NIH Director’s Biomedical Research Workforce Innovation Award: Broadening Experiences in Scientific Training (BEST),” therein soliciting applications from institutions to experiment with novel programs that would provide doctoral and postdoctoral trainees with opportunities and expertise to explore the myriad of careers available to them (https://grants.nih.gov/grants/guide/rfa-files/rfa-rm-12-022.html). Over a 2-year period, BEST grants were awarded to 17 institutions [13, 14] with extensive career data collection and reporting required.

Several BEST-funded and non-BEST institutions have begun to collect and report data (often on program Web sites) on doctoral and/or postdoctoral alumni career outcomes [4, 15] and are publically reported. Examples include: http://web.stanford.edu/dept/pres-provost/irds/phdjobs, https://medschool.vanderbilt.edu/bret/igp-qcb-admissions-and-outcomes-data, http://bbsp.unc.edu/prospective-students/outcomes/ and detail workforce sectors in which the alumni were employed, types of careers they were pursuing, and the job functions in which they were engaged, adapted generally from myIDP [16]. The BEST community piloted the collection of career outcomes data to demonstrate to federal funding agencies and policymakers the wide reach and tremendous impact of biomedical research trainees in driving advances in biomedical science and research both inside and outside of traditional academic environments.

However, when the BEST programs began this project, there was no commonly held taxonomy of career outcomes that had been agreed upon that might be used to:

- Collate and compare outcomes between institutions;
- Modify institutional recruitment and training structures and programs guided by known career outcomes;
- Modify training to respond to emerging technologies in the biomedical sciences;
- Provide potential trainees with data to help them to select institutions and mentors that match their career aspirations;
- Disseminate and test the taxonomy with other disciplines.

In 2017, several members of the BEST consortium, Association of American Medical Colleges (AAMC) Graduate Research Education and Training (GREAT) group, and Rescuing Biomedical Research (RBR), collectively proposed a 3-tier taxonomy. The first tier of this taxonomy includes 5 career sectors, the second tier includes 5 career types, and the third tier defines 24 job functions (Table 1). A number of institutions within the BEST consortium have piloted the use of this taxonomy for their institutional doctoral alumni career outcomes and several have agreed to contribute their institutional data to populate the aggregated database emerging from this taxonomy.

**Table 1:** Three-tier career taxonomy

Tier 1: career sectors	Tier 2: career types	Tier 3: job functions
Academia	Primarily research	Administration
Government	Primarily teaching	Business development, consulting, and strategic alliances
For-profit	Science-related	Clinical research management
Nonprofit	Not-related to science	Clinical services
Other	Further training or education	Data science, analytics, and software engineering
		Entrepreneurship
		Faculty: nontenure track
		Faculty: tenured/tenure track
		Faculty: track unclear or not applicable
		Full-time teaching staff/instructor
		Group leader (research)
		Healthcare provider
		Intellectual property and law
		Part-time teaching staff/adjunct
		Postdoctoral research
		Regulatory affairs
		Research staff or technical director
		Sales and marketing
		Science education and outreach
		Science policy and government affairs
		Science writing and communication
		Technical support and product development
		Completing further education or training
		Other
		Deceased/retired

There are many potential uses for the data collected through implementation of this taxonomy. For example, data collected by BEST institutions have already illustrated that alumni are widely and extensively employed in diverse career sectors and types utilizing job functions with the potential to contribute to scientific advancements.

We encourage all biomedical doctoral and postdoctoral training institutions to consider adopting this common taxonomy to report outcomes of their trainees and explore the utility of the data within their own institution. Suggestions for how to use and implement the taxonomy can be viewed on the Web site of the BEST consortium [17].

It is important to note that this taxonomy is suitable for use across disciplines and beyond the biomedical fields. This is particularly important in institutions where resource-strapped graduate schools or other entities are currently tasked with collecting alumni career outcomes data from multiple disciplines using different taxonomies. For instance, at Wayne State University, consensus was reached on the applicability and use of Tier 1 and Tier 2 across disciplines beyond biomedical sciences, including other life sciences, physical sciences, social and behavioral sciences, and the humanities. Tier 3 includes more specific job functions and can be adapted to fit disciplinary needs.

One existing approach for gathering data about Ph.D. career pathways is the Council of Graduate Schools’ (CGS) PhD Career Pathways project, which has brought together 59 universities to gather data on the career aspirations of current Ph.D.s and the career outcomes of Ph.D. alumni. This effort, funded by the National Science Foundation (NSF) and the Andrew W. Mellon Foundation, is designed to help institutions gather data about their Ph.D.s across all broad fields. At a meeting organized by CGS in December 2017 attended by some members of the BEST consortium, the AAMC GREAT group, the RBR group, and the NIH, the organizations present explored ways to reduce the survey burden on institutions that may be participating in a number of these efforts, including CGS’s effort, and agreed that there is potential for a crosswalk between Tiers 1 and 2 of the BEST taxonomy. The unified adoption of Tiers 1 and 2 will enrich our understanding of commonalities and differences in the types of careers that Ph.D. recipients across all disciplines pursue. This is relevant given that the shift away from academic careers is occurring in all disciplines and is not confined to the biomedical sciences alone. As programs shift their training paradigms accordingly and provide trainees with opportunities to explore and succeed in all careers, the biomedical training community can learn and share best practices with trainers in other disciplines.

## Conclusions

This 3-tier taxonomy is a valuable resource for institutions committed to greater transparency in reporting Ph.D. and postdoctoral career outcomes. This transparency has been emphasized by the BEST grant awardees as well as by presidents and chancellors of 10 leading institutions [18]. Notably, a number of institutions, including those of some of the authors of this manuscript (University of North Carolina-Chapel Hill, Vanderbilt, and Wayne State University), had already been reporting their data publicly for several years on Web sites and in publications using a very similar taxonomy. A single unified taxonomy permits institutions not only to be transparent in reporting their own institutional data but also to lend greater power to a central repository. These aggregated data could be analyzed and reported to federal funding agencies, the public, science policymakers, and trainees so that all stakeholders can understand and appreciate the breadth of careers that Ph.D.-trained scientists pursue. The fact that our alumni are making significant impacts in academia and in all areas of the workforce such as industry, business, entrepreneurship, government, policy, and communication needs to be broadly disseminated to emphasize the societal and financial impact of doctoral and postdoctoral training.
